# Genetic Evidence for Involvement of Neuronally Expressed S1P_1_ Receptor in Nociceptor Sensitization and Inflammatory Pain

**DOI:** 10.1371/journal.pone.0017268

**Published:** 2011-02-17

**Authors:** Norbert Mair, Camilla Benetti, Manfred Andratsch, Michael G. Leitner, Cristina E. Constantin, Maria Camprubí-Robles, Serena Quarta, Wolfgang Biasio, Rohini Kuner, Ian L. Gibbins, Michaela Kress, Rainer V. Haberberger

**Affiliations:** 1 Division of Physiology, Department of Physiology and Medical Physics, Innsbruck Medical University, Innsbruck, Austria; 2 Department of Pharmacology, University Heidelberg, Heidelberg, Germany; 3 Flinders Medical Science and Technology, Centre for Neuroscience, Flinders University, Adelaide, Australia; Chiba University Center for Forensic Mental Health, Japan

## Abstract

Sphingosine-1-phosphate (S1P) is a key regulator of immune response. Immune cells, epithelia and blood cells generate high levels of S1P in inflamed tissue. However, it is not known if S1P acts on the endings of nociceptive neurons, thereby contributing to the generation of inflammatory pain. We found that the S1P_1_ receptor for S1P is expressed in subpopulations of sensory neurons including nociceptors. Both S1P and agonists at the S1P_1_ receptor induced hypersensitivity to noxious thermal stimulation *in vitro* and *in vivo*. S1P-induced hypersensitivity was strongly attenuated in mice lacking TRPV1 channels. S1P and inflammation-induced hypersensitivity was significantly reduced in mice with a conditional nociceptor-specific deletion of the S1P_1_ receptor. Our data show that neuronally expressed S1P_1_ receptors play a significant role in regulating nociceptor function and that S1P/S1P_1_ signaling may be a key player in the onset of thermal hypersensitivity and hyperalgesia associated with inflammation.

## Introduction

Thermal and mechanical hypersensitivity, and ongoing pain, are distressing symptoms common to many inflammatory conditions. The inflammatory process involves a multitude of cellular interactions between local tissue components, invading cells of the immune system and the peripheral terminals of nociceptive neurons (nociceptors). Bioactive lipids are strongly associated with inflammation including chronic inflammatory diseases such as relapsing multiple sclerosis (MS) or rheumatoid arthritis (RA) [1]–[4]. In particular, the sphingolipid sphingosine-1-phosphate (S1P) has turned out to be a multifaceted immune modulator acting intracellularly as a second messenger molecule or extracellularly in an autocrine or paracrine manner [5]–[7].

Extra- and intracellular levels of S1P are tightly regulated by sphingosine kinases (SphKs) and S1P degrading enzymes [8], [9]. Activation of SphK1 is the key event in elevating S1P levels [10]–[12]. S1P is deactivated by S1P-phosphatases or degraded by S1P-lyase [13]–[15]. Mice deficient in both SphK1 and SphK2 are not viable, indicating an essential cellular requirement for S1P in addition to its role in inflammation [16].

Immune cells, epithelia and neurons can generate S1P when stimulated by chemo-attractants like tumor necrosis factor alpha (TNFα) or nerve growth factor (NGF) [10], [17]. They release S1P via a specific transporter, the multidrug resistance-associated protein ABCC1 [6]. Although S1P in blood plasma may reach up to micromolar concentrations, it is largely bound to plasma [18]–[20]. Systemic S1P concentrations are increased in RA patients [21]. Moreover, high concentrations of free S1P can arise locally at inflammation sites [6], [22]. In rheumatoid synovium S1P/S1P_1_ receptor signaling appears to be a key regulator of the local immune response [2], [4], and increasing evidence supports a critical role of S1P in autoimmune processes in RA [23]. In the general population, RA is a common cause of disability with a prevalence of 1%, and more than 95% of the patients report moderate to severe ongoing pain [24]. Advances in the treatment of RA with immunosuppressive therapies promise to improve the patients' quality of life [25], [26]. In a murine model of RA, the novel immune modulator and S1P receptor ligand FYT720 inhibits arthritis [27].

These observations led us to hypothesize that S1P may not only regulate local immune cells, synoviocytes and osteoclasts [23], [28], [29], but may also act on nociceptors to cause inflammatory pain. We discovered that S1P_1_ receptors were expressed by a subpopulation of nociceptive neurons. We found evidence that S1P induced significant hypersensitivity *in vitro* and *in vivo.* Moreover, we generated mice lacking the S1P_1_ receptor in neurons expressing the nociceptor-specific Na_v_1.8 promoter. These mice were largely protected from S1P-induced hypersensitivity. Based on these results, we conclude that S1P plays a critical role in regulating nociceptor sensitivity and that this action is largely mediated by neuronally expressed S1P_1_ receptors.

## Results

### S1P-induced hypersensitivity to heat stimuli and sensitization of nociceptor responses

**Figure 1:**
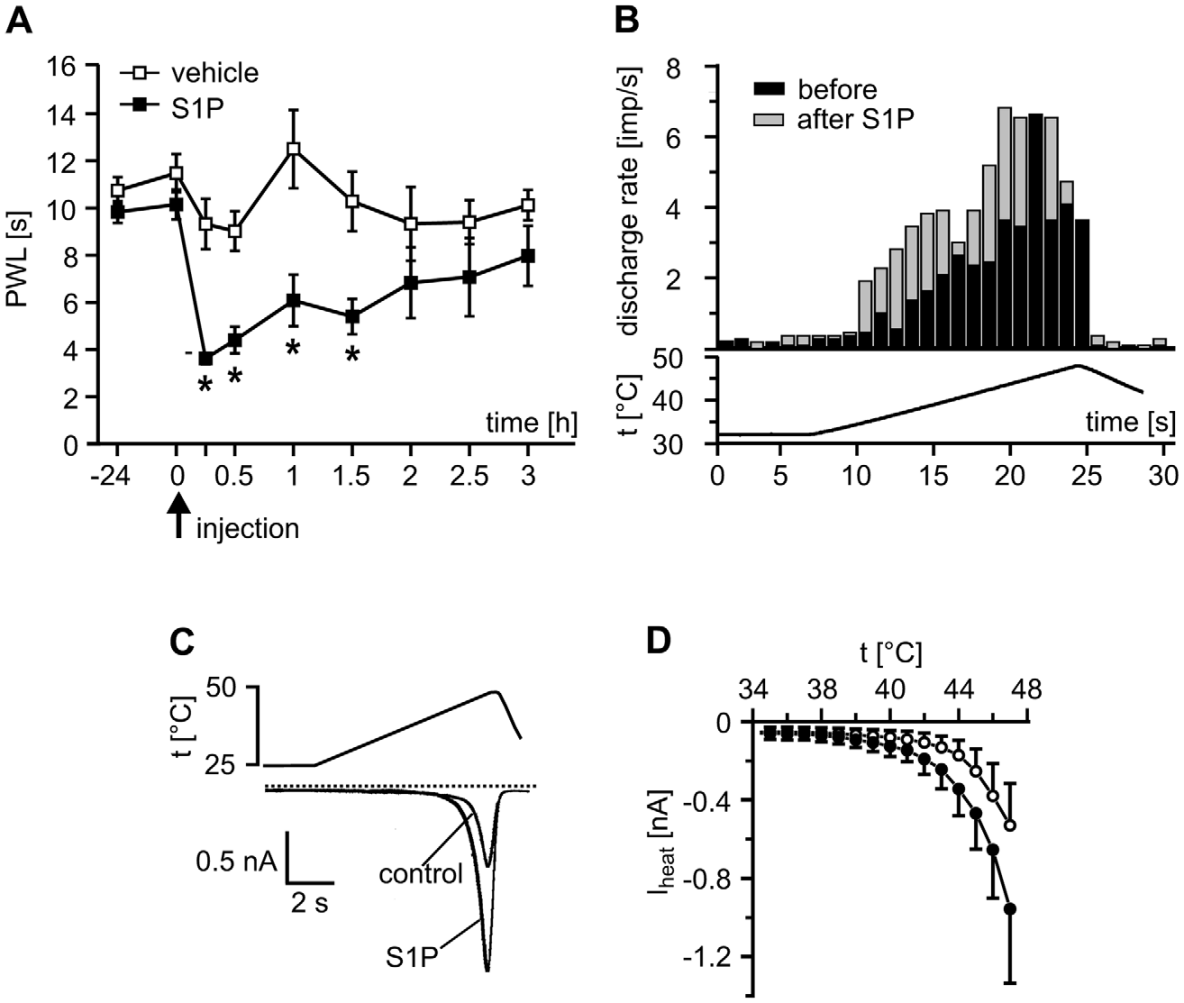
S1P-induced sensitization of heat pain behavior, nociceptor neuron discharge *in vitro* and heat-activated ionic currents. (A) Injection of S1P into the paw skin (5 µl of a 100 µM S1P solution in PBS) but not vehicle (n = 10, n.s.) induced a significant transient drop in paw withdrawal latencies (PWL) in response to heat stimulation from 10.15±0.63 to 3.62±0.24 s (n = 10, *p<0.05; ANOVA). Heat sensitization fully recovered to baseline within three hours. (B) Discharge activity of single primary nociceptive neurons *in vitro* significantly increased from 2.03±0.39 before (black columns) to 3.21±0.50 Imp/s (grey columns) after the receptive fields of the fibers were exposed to 1 µM S1P for 5 min (n = 11, p<0.05; Wilcoxon matched pairs test). (C) After conditioning stimulation with S1P, the heat-induced current of a dorsal root ganglion neuron exhibited increased peak amplitudes and was activated at a lower temperature compared with control. (D) Temperature-current plots of four neurons stimulated with a ramp-shaped heat stimulus with a linear rise of temperature from room temperature to 50°C before (open circles) and after conditioning stimulation with S1P (filled circles, threshold temperature).

The latency of the withdrawal reaction in response to radiant heat is a reliable parameter to monitor changes in sensitivity to painful heat stimuli in rodents [30]. Heat withdrawal latencies were determined in wt C57BL/6J mice that received intracutaneous injections of S1P (5 µl, 100 µM) in phosphate-buffered saline or vehicle only. Within fifteen minutes after injection of S1P but not vehicle, mean withdrawal latencies decreased significantly (p<0.05; Mann-Whitney U-test; Fig. 1A). Hypersensitivity to heat stimulation was maintained for more than two hours; after three hours, paw withdrawal latencies were no longer significantly different from vehicle-injected sites.

Extracellular recordings of single unit activity from unmyelinated primary afferents in an isolated skin-nerve preparation showed that S1P increased responsiveness of nociceptive neurons to heat stimulation. When the receptive fields of polymodal C-fibers were exposed to a conditioning stimulus of S1P (1 µM, 5 min), the average number of action potentials in response to a standard heat stimulus was significantly augmented compared with the control response. Furthermore, thermal thresholds for C-fiber activation were significantly lower after S1P application (p<0.05, n = 7; Student's paired t-test; Fig. 1B).

### S1P-induced potentiation of heat and capsaicin-activated excitatory inward currents

The isolated skin-nerve preparation excludes the possibility of chemotactic invasion of immune cells, and the density of resident macrophages in healthy skin and tissue is low. To test if S1P affected nociceptive neurons directly, we performed whole-cell patch clamp measurements of heat-evoked excitatory inward currents (I_heat_) in capsaicin-sensitive neurons acutely isolated from mouse dorsal root ganglia (DRG). S1P caused a significant dose-dependent increase of I_heat_ peak amplitudes and threshold activation temperatures of I_heat_ were reduced by approximately 2°C (Fig. 1C, D). The potentiation of I_heat_ fully recovered within 6 min suggesting relatively short-term modulation of a thermosensitive ion channel by S1P.

**Figure 2:**
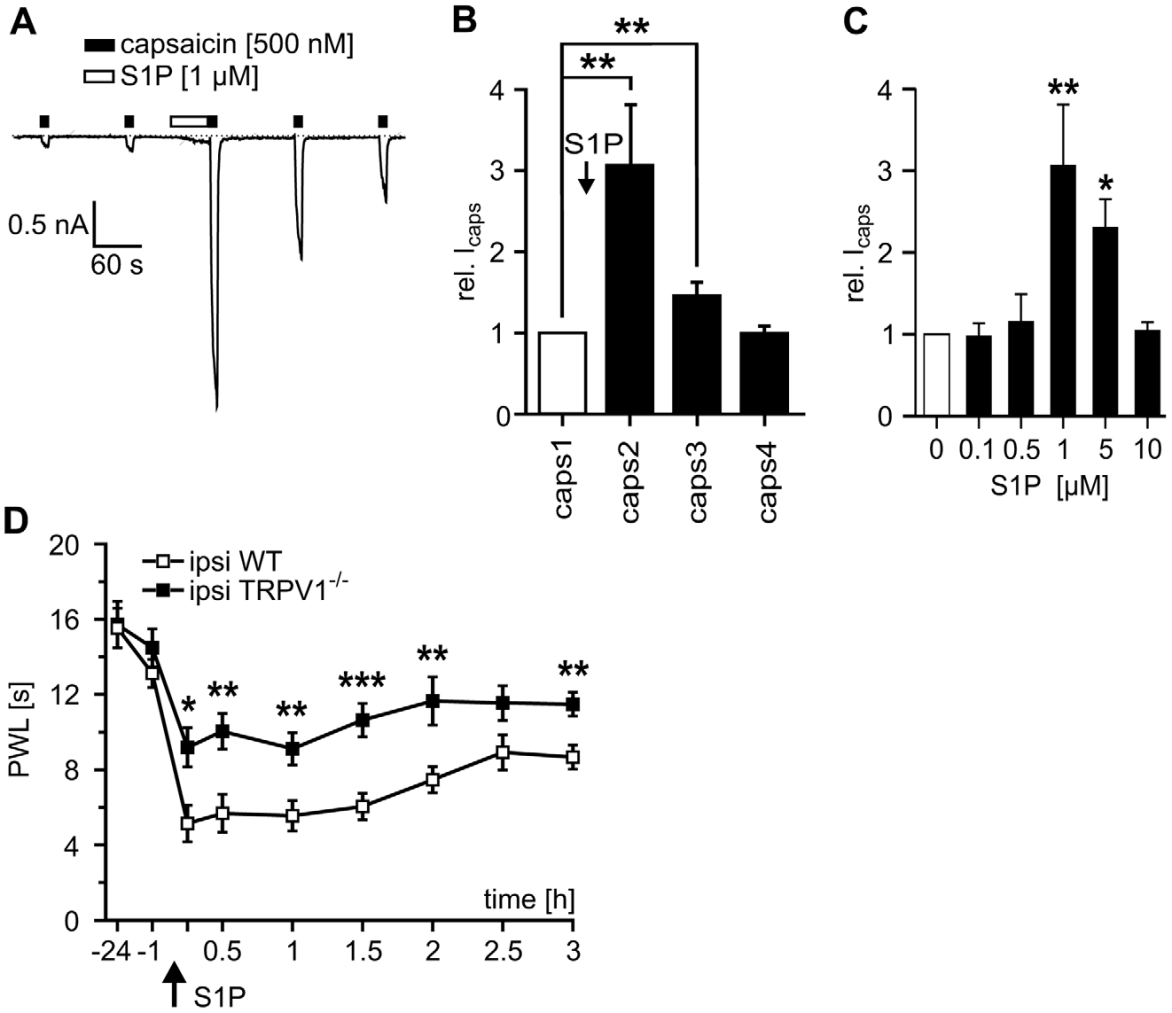
S1P-induced hypersensitivity largely depends on TRPV1 channels. (A) Currents elicited by the TRPV1 activator capsaicin (500 nM for 10 s, black boxes) were strongly facilitated after S1P (1 µM, 60 s, open box). (B) Average responses to repeated stimulation with 500 nM capsaicin before and after S1P (duration 3 s, interval 120 s). I_caps_ significantly increased by a factor of 3.1±0.74 after conditioning S1P application (n = 9, p<0.01; Wilcoxon matched pairs test) and fully recovered within 4 min. (C) Dose-response relationship for S1P-induced facilitation of I_caps_. A maximum effect was observed at a S1P concentration of 1 µM with a half-effective dose ED50 of 0.55 µM. At concentrations exceeding 1 µM the sensitizing effect of S1P became less pronounced. (D) The S1P-induced reduction in paw withdrawal latencies was significantly attenuated but not fully abolished in TRPV1 null mutant mice (filled squares) as compared to wt littermates (open squares, n = 0, *p<0.05, **p<0.01, *** p<0.001; ANOVA).

One important thermosensitive ion channel in nociceptive neurons is the capsaicin-activated transient receptor potential vanilloid receptor, TRPV1 [31]. We found that S1P augmented capsaicin-induced currents (I_caps_) with a half maximal effective concentration of 0.55 µM under controlled single cell perfusion conditions (500 nM capsaicin; p<0.01; n = 7; Wilcoxon matched pairs test Fig. 2A, B). The maximum increase occurred at 1 µM S1P; lower and higher concentrations were less effective. S1P-induced heat hyperalgesia was significantly ameliorated, but not fully abolished, in TRPV1^−/−^ mice compared with wt littermates (Fig. 2D).

### S1P_1_ receptor mRNA and protein expressed in distinct neuron populations

**Figure 3:**
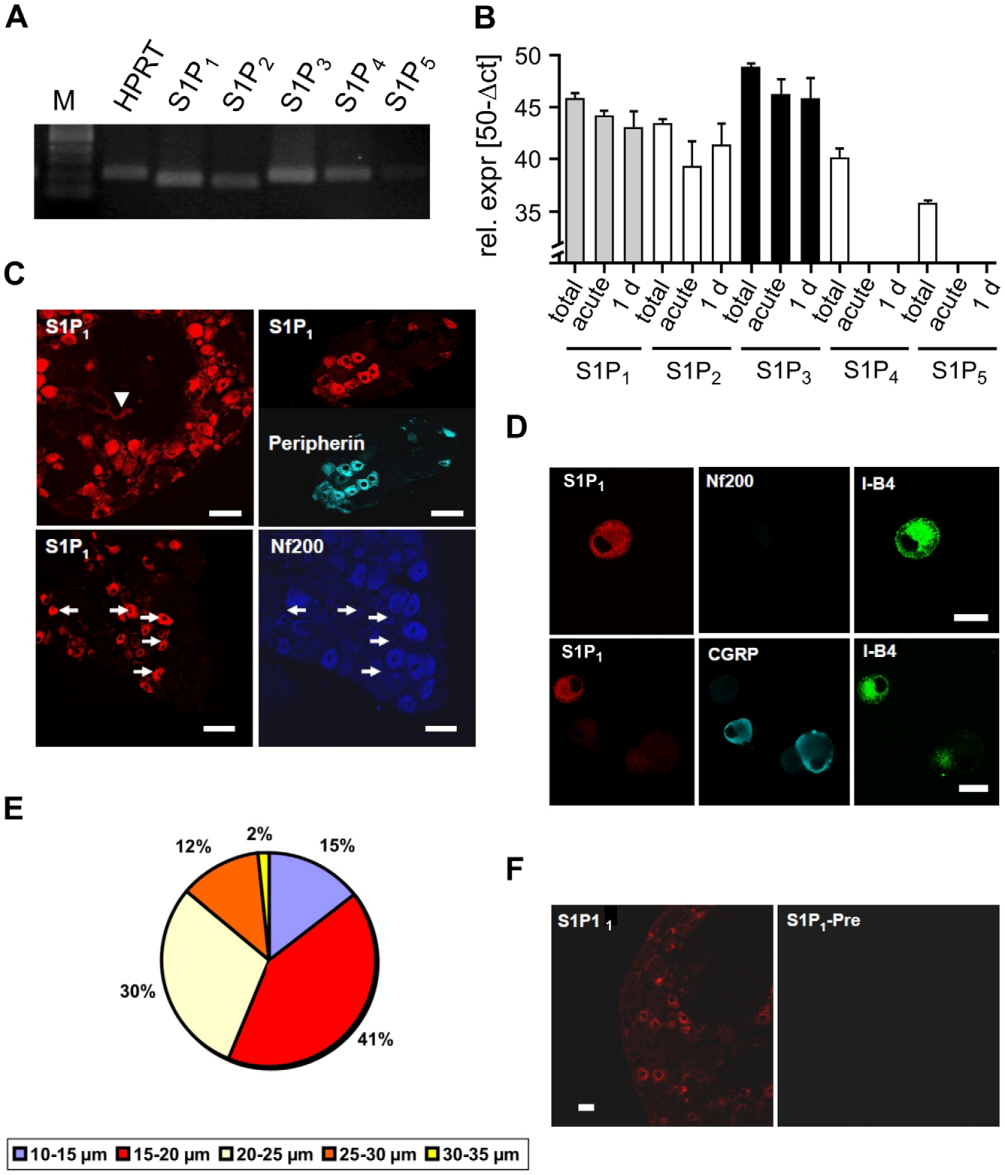
Expression of S1P_1_ receptors in sensory neurons. (A) S1P receptor mRNA expression was detected with reverse transcription PCR in DRG explants. (B) Quantitative real-time PCR revealed expression of S1P_1_, S1P_2_ and S1P_3_ mRNA in DRG explants (total), acutely isolated neurons (acute) and 1-day-old cultures (1 d) (n = 5 experiments). In contrast, S1P_4_ and S1P_5_ mRNA levels were lower in DRG explants and absent in isolated neurons. (C) Immunoreactivity for S1P_1_ was present in neurons and intraganglionic capillaries (arrowhead). S1P_1_-IR was colocalized with immunoreactivity for peripherin, whereas S1P_1_-IR was absent in NF200-positive neurons. Scale bars  = 50 µm. (D) S1P_1_ receptor colocalized with the small neuron marker I-B4 in the vast majority of cultured neurons but usually not with CGRP or Nf200, a marker for myelinated neurons (n = 4 experiments, scale bars  = 20 µm). (E) Size distribution of S1P_1_-IR positive neurons revealed that S1P_1_-IR expressing cells are amongst the small diameter neurons (n = 6 experiments, 304 neurons). Only 2% of S1P_1_-IR+ neurons had diameters >20 µm. (F) Expression of S1P_1_ immunoreactivity was absent after preabsorption of the antibodies with the corresponding peptide.

To date, five metabotropic S1P_1–5_ receptors for S1P have been identified which are members of the *edg* (endothelial differentiation gene) family of G-protein coupled receptors (GPCRs) [32]–[35]. To elucidate which S1P receptors were relevant for the regulation of thermal sensitivity in sensory neurons, we performed receptor mRNA expression profile analyses in DRG explants, in acutely dissociated neurons, and in primary DRG neuron enriched cultures. Quantitative PCR showed the following rank order of relative receptor mRNA expression for DRG explants: S1P_3_ > S1P_1_ > S1P_2_ > S1P_4_ > S1P_5_ (Fig. 3A, B). These explants contain neurons and non-neuronal cells including satellite cells, myelinating and non-myelinating Schwann cells, resident immune cells, endothelial cells and vascular immune cells [36]. S1P receptors have been found expressed in most of these cell types (for review see [37]. However, in preparations enriched with acutely isolated sensory neurons (<1% non-neuronal cells) and in 1-day-old neuron-enriched cultures mRNAs for S1P_1_, S1P_2_ and S1P_3_ but not S1P_4_ and S1P_5_ receptors were expressed. The relative expression levels of mRNA for S1P_1–3_ receptors were similar in acutely dissociated neurons and in 1-day-old cultures (Fig. 3A, B). Together this suggests that S1P_1_, S1P_2_ and S1P_3_ receptor subtypes were expressed in sensory neurons whilst S1P_4_ and S1P_5_ subtypes were not. We conclude, therefore, that in the explant preparations, mRNAs for S1P_4_ and S1P_5_ receptors were probably derived from non-neuronal cells within the DRG. Most nociceptive neurons are small to medium-sized peripherin-positive neurons [38]. They include glial-derived neurotrophic factor (GDNF)/protein typrosine kinase receptor (c-RET) dependent neurons with affinity of the isolectin B4 (I-B4+) and NGF/tyrosine kinase A (TrkA) dependent neurons that contain the neuropeptides calcitonin-gene related peptide (CGRP) and substance P [39]. Immunoreactivity for S1P_1_ receptor was found predominantly in small peripherin-IR neurons in DRG sections (Fig. 3C, D, E, F). In sections and neuron cultures, S1P_1_ immunoreactivity predominantly occurred in small I-B4+, Nf200-negative neurons. A smaller group of CGRP-IR neurons also displayed immunoreactivity for S1P_1_ (Table 1). Based on this co-expression profile, we predicted that the actions of S1P on nociceptor function were most likely mediated by the S1P_1_ receptor.

**Table 1:** Percentages of immunoreactive neurons (n = 6 mice, n = 1129 neuronal profiles).

	S1P_1_	
	% of total number	diameter [µm]
Immunoreactive neurons/DRG section,	35.8±8.1	18.6±0.8
Colocalization with CGRP+	6. 8±2.3	17.5±0.9
Colocalization with Nf200+	<1%	

### Agonists at S1P_1_ receptor induced hypersensitivity

**Figure 4:**
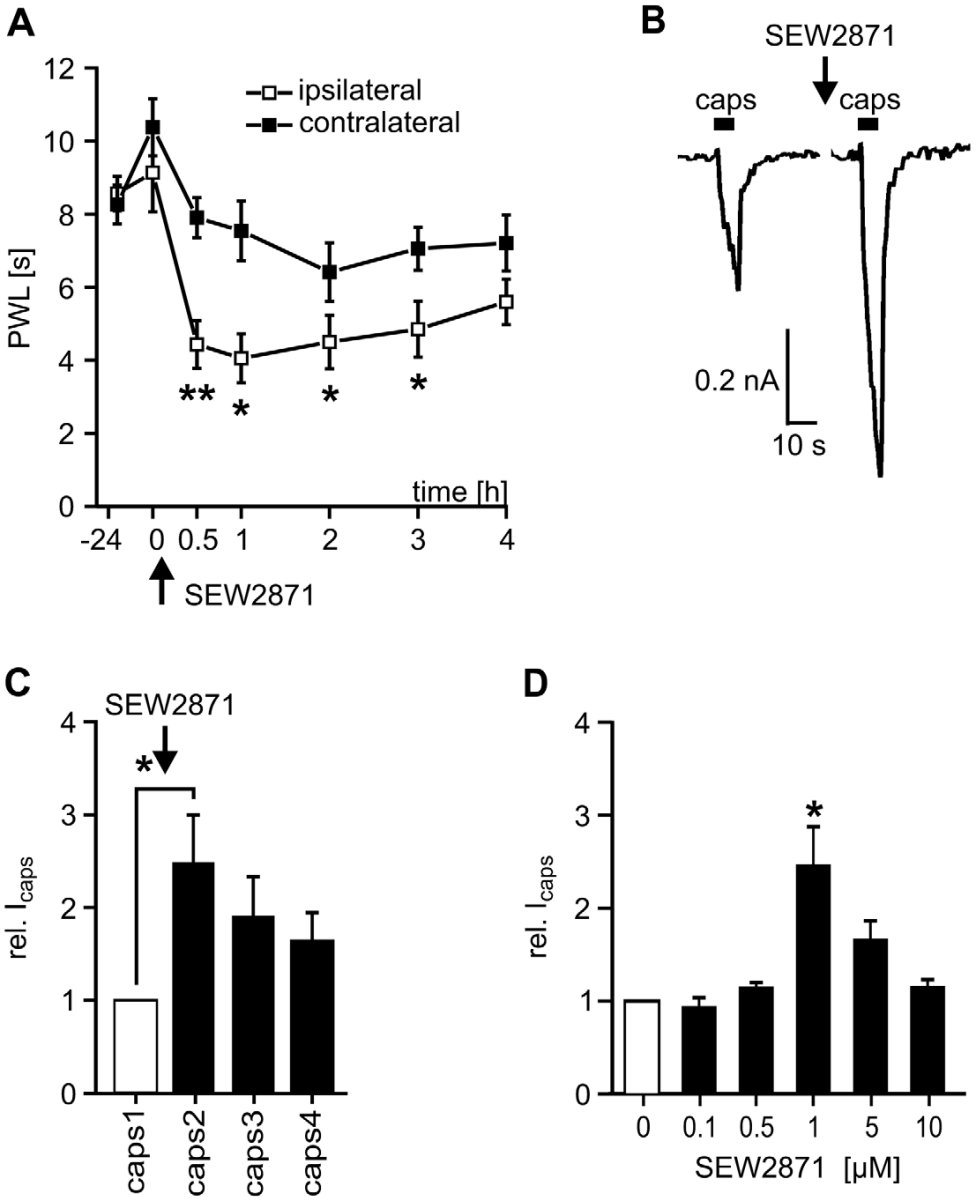
Significance of S1P_1_-mediated heat hypersensitivity. (A) Injection of the S1P_1_-selective agonist SEW2871 induced a significant reduction of PWL in C57BL/6J mice (100 µM, 5 µl, n = 8, *p<0.05, **p<0.01; ANOVA). (B) Conditioning stimulation of DRG neurons with the S1P_1_ agonist SEW2871 (1 µM) induced an increase in I_caps_ in an isolated DRG neuron. (C) SEW2871 (1 µM) significantly elevated average responses to consecutive capsaicin stimuli (1 to 4, 3 s at 120 s intervals) by a factor of 2.47±0.41 compared with control peak amplitudes (n = 6, *p<0.05; Wilcoxon matched pairs test). (D) Dose-response relationship of SEW2871 sensitization of I_caps_. The ED50 was 0.56 µM and the maximum effect was observed with 1 µM SEW2871, the same concentration that gave the highest responses for S1P.

SEW2871 was developed as a selective agonist of S1P_1_ receptors [40]. Intracutaneous administration of SEW2871 (5 µl, 100 µM) induced heat hypersensitivity *in vivo,* and mean paw withdrawal latencies to noxious heat stimulation dropped significantly to a degree similar to that obtained for S1P injection (n = 8, p<0.05; ANOVA; Fig. 4A). The time course of SEW2871-induced heat hypersensitivity was similar to that of S1P and recovered slowly two hours after injection. In acutely isolated neurons, I_caps_ was potentiated by SEW2871 with a time course and half maximal concentration similar to those seen in response to S1P (Fig. 3B–D). SEW2871 facilitated I_caps_, and peak current amplitudes transiently increased in a dose-dependent manner with the maximum effect occurring at 1 µM (2.46±0.50 fold, p<0.05, n = 6; Wilcoxon matched pairs test; Fig. 4D). These similarities support our hypothesis that nociceptor sensitization predominantly occurred through activation of the S1P_1_ receptor.

### Conditional and specific deletion of S1P_1_ receptors in sensory neurons

**Figure 5:**
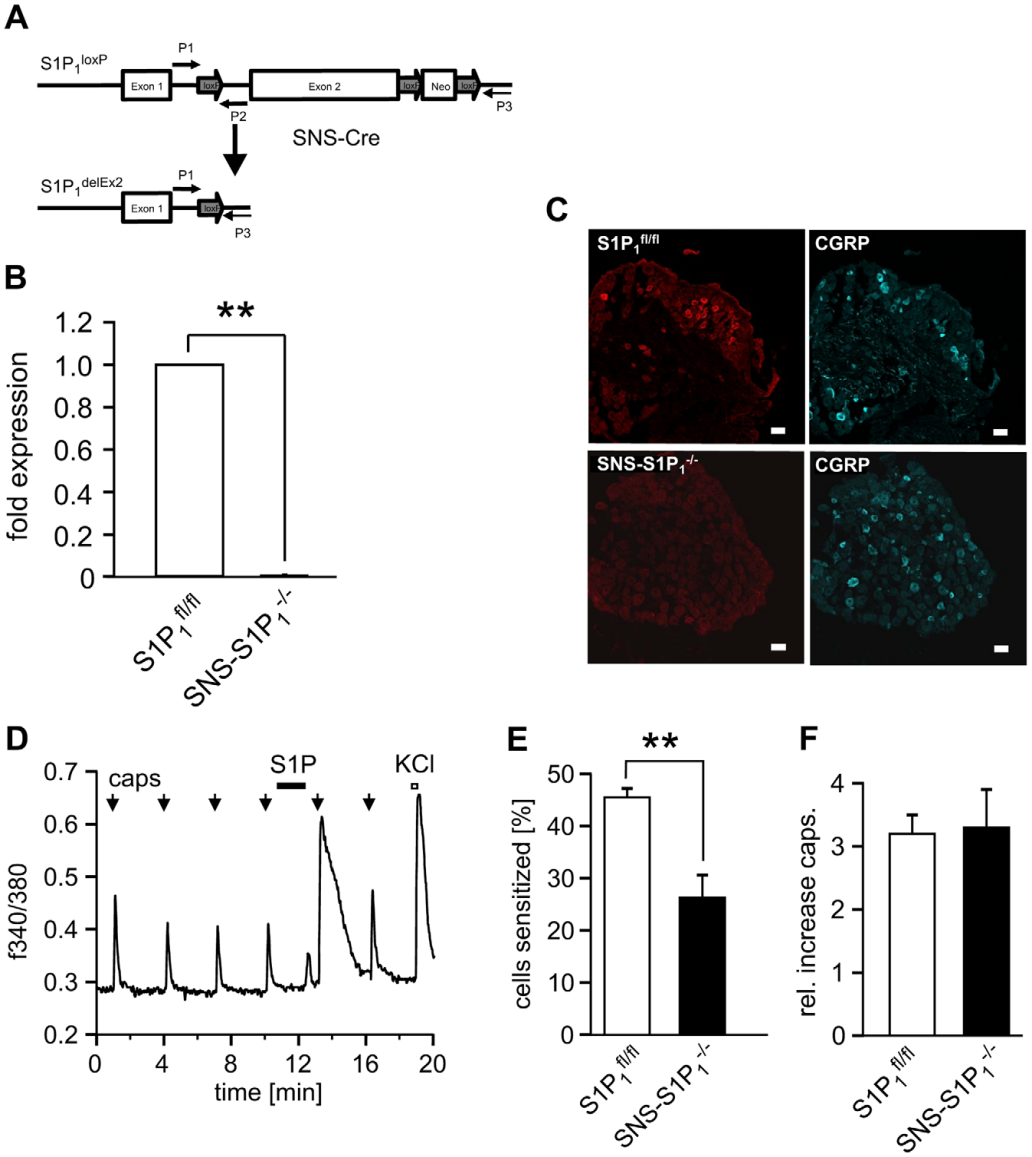
SNS-S1P_1_^−/−^ mice are largely protected from S1P-induced hypersensitivity. (A) Deletion of exon 2 in nociceptive neurons with the SNS-Cre recombination methods in *SNS-Cre:S1P_1_^fl/fl^* (SNS-S1P_1_^−/−^) mice. (B) Taqman®-PCR analysis of DRG explants revealed an almost complete absence of S1P_1_ mRNA (n = 10) in SNS-S1P_1_^−/−^ mice in comparison to control S1P_1_^fl/fl^ mice (n = 9, **p<0.01; Mann-Whitney U-test). (C) S1P_1_ receptor immunoreactivity is expressed in a subpopulation of small size sensory neurons in DRG sections obtained from S1P_1_^fl/fl^ but not in SNS-S1P_1_^−/−^ mice. There is no difference in the expression profile of CGRP immunoreactivity. (D) Example of a neuron that responded to capsaicin (arrows) with calcium transients. S1P itself induced a brief transient which recovered immediately and the following response to capsaicin was strongly increased. (E, F) The percentage of neurons responding to S1P with an increase in capsaicin-induced calcium transients was significantly reduced in SNS-S1P_1_^−/−^ mice compared to S1P_1_^fl/fl^ mice.

Mice deficient of the S1P_1_ receptor die 12.5 days after conception or birth, depending on the genetic background [41]. Therefore, conditional knockout mice lacking S1P_1_ selectively in nociceptive neurons of the dorsal root ganglion (DRG) were generated using the Cre recombinase loxP strategy and conditional removal of exon 2 (Fig. 5A) which encodes the entire coding region of S1P_1_ [42]. This was achieved by mating homozygous mice carrying the *loxP*-flanked (floxed) S1P_1_ (S1P_1_^fl/fl^) [42] with a mouse line expressing Cre recombinase under the transcriptional control of the nociceptor-specific Na_v_1.8 gene (SNS-Cre) [43]. In SNS-Cre mice, gene recombination reportedly occurs in around 90% of small diameter (≤28 µm) nociceptive sensory neurons, commences at birth and does not affect gene expression in the spinal cord, brain or any other organs in the body [43]–[45]. Sequence comparisons of PCRs using primers P1 and P3 of cDNA from S1P_1_^+/+^, S1P_1_^fl/fl^ and SNS-S1P_1_^−/−^ DRG confirmed that DRG neurons from SNS-S1P_1_^−/−^ mice lacked mRNA for S1P_1_ receptors. Moreover, antibodies directed against the S1P_1_ receptor [46] showed immunoreactivity to S1P_1_ receptor protein in DRG from S1P_1_^fl/fl^ but not SNS-S1P_1_^−/−^ mice (Fig. 5B, C). For quantitative analysis, we recorded capsaicin-induced calcium transients in DRG neurons before and after conditioning stimulation with S1P which regularly induced brief calcium transients itself. After S1P the capsaicin responses were strongly facilitated (Fig. 5D). The number of neurons sensitized to capsaicin after conditioning stimulation with S1P was significantly lower in SNS-S1P_1_^−/−^ mice compared with S1P_1_^fl/fl^ mice (p<0.01; Student's unpaired t-test; Fig. 5E) but the magnitude of the increased response in sensitized neurons was similar for both strains (Fig. 5F).

### Mice lacking S1P_1_ in Na_v_1.8 expressing nociceptors show reduced S1P-induced hypersensitivity

**Figure 6:**
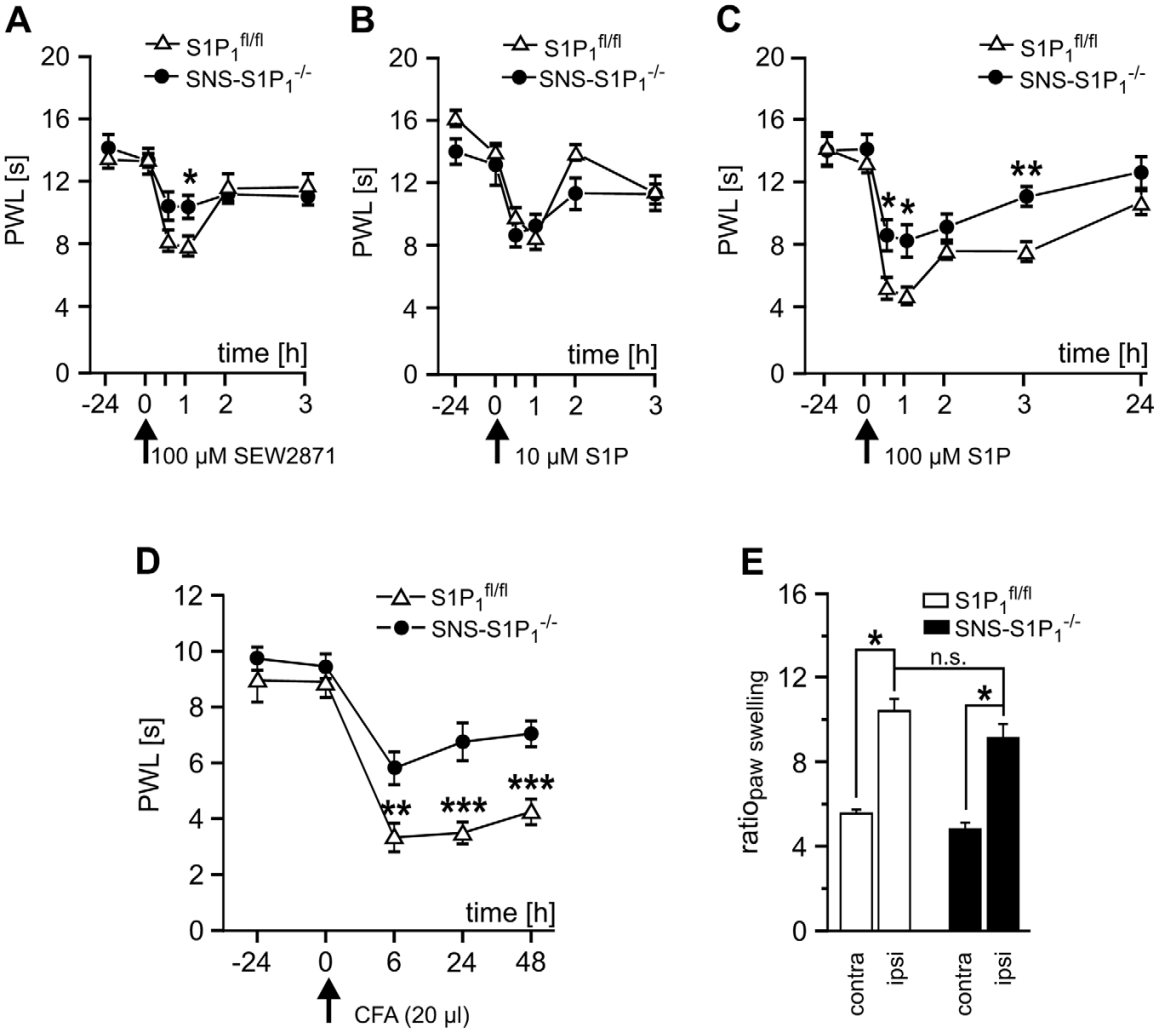
Reduced thermal hypersensitivity in S1P_1_^−/−^mice. (A) Injection of the S1P_1_ agonist SEW2871 induced a significant transient decrease in paw withdrawal latencies in S1P_1_^fl/fl^ (n = 9) which was significantly less pronounced than in SNS-S1P_1_^−/−^ mice (n = 10, *p<0.05; ANOVA). (B, C) While only a minor reduction of paw withdrawal latencies was observed in both mouse strains with local low dose S1P injection, we observed a significant decrease in paw withdrawal latencies in S1P_1_^fl/fl^ mice (n = 7) which was similar to wt. In SNS-S1P_1_^−/−^ mice the degree of hypersensitivity was significantly ameliorated in comparison to S1P_1_^fl/fl^ mice (n = 9, *p<0.05, ** p<0.01; ANOVA). (D) CFA (20 µl) injection into the plantar hindpaw induced a pronounced decrease of PWL which was significantly attenuated in S1P_1_-Cre mice (p<0.05, n = 4; ANOVA). (E) Paw swelling was similar in SNS-S1P_1_^−/−^ and S1P_1_^fl/fl^ mice (n = 4).

SNS-S1P_1_^−/−^ mice were viable and did not show any obvious deficits in spontaneous behavior, motor capabilities or breeding. Baseline mechanical and thermal thresholds of cutaneous sensory neurons were similar to S1P_1_^fl/fl^ littermates. In S1P_1_^fl/fl^ mice, intracutaneous injection of the S1P_1_ receptor agonist SEW2871 resulted in a transient and significant drop in paw withdrawal latencies which fully recovered after two hours (Fig. 6A). In SNS-S1P_1_^−/−^ mice, the change in heat sensitivity was equal to vehicle injection. Lower doses of S1P itself induced a short hypersensitivity that was similar in S1P_1_^fl/fl^ and SNS-S1P_1_^−/−^ mice (Fig. 6B). At higher doses, S1P induced similar reductions in withdrawal latencies in S1P_1_^fl/fl^ and C56BL/6J wt mice, however, the decrease in paw withdrawal latency was significantly smaller in SNS-S1P_1_^−/−^ (Fig. 6C). Moreover, in SNS-S1P_1_^−/−^ mice the decrease of paw withdrawal latency after CFA-induced inflammation was significantly reduced in comparison to S1P_1_^fl/fl^ (5.8±0.6 s vs. 3.3±0.5 s, 6 h after CFA injection; n = 8; ANOVA repeated measures and Tukey post hoc test, genotype: F(1,79)  = 24.9; p<0.001, time points: F(4,79)  = 39.7; p<0.001; genotype × time points: F(4,79)  = 2.82; p = 0.033; Fig. 6D). The degree of inflammation (shown as ratio_paw swelling_; Fig. 6E) was similar in both groups. The degree of mechanical hypersensitivity after S1P injection was not significantly different from controls in SNS-S1P_1_^−/−^ mice (9.5±1.1 mN before vs. 3.7±0.8 mN, 6 h after CFA injection; n = 7; ANOVA). Based on the evidence we found, we propose that S1P/S1P_1_ receptor signaling in nociceptive primary afferents could be relevant for inflammatory pain.

## Discussion

This study revealed a significant role of S1P in increasing nociceptor sensitivity to noxious thermal stimuli *in vivo* and *in vitro*. Specifically, both S1P and agonists selective for S1P_1_ receptors acutely sensitized peripheral nociceptive nerve terminals to noxious heat. They also augmented heat or capsaicin-activated inward currents in nociceptive neurons resulting in their increased excitability. S1P-induced hypersensitivity to thermal stimulation was strongly reduced in mice lacking either TRPV1 channels or neuronally expressed S1P_1_ receptors.

Heat-activated currents in sensory neurons were activated at lower temperatures in response to S1P and nociceptors responded earlier to thermal stimuli applied to the receptive field since the mechanism for transducing noxious thermal stimuli became more efficient. Moreover, treatment with S1P increased the excitability of sensory neurons via GPCR-dependent enhancement of TTX-resistant sodium inward currents and inhibition of potassium outward currents [35]. In combination, these actions of S1P on nociceptors are consistent with a significant role in inflammatory hyperalgesia.

Primary afferent nociceptors fall into at least two distinct subclasses: those expressing neuropeptides, especially CGRP; and those non-peptidergic neurons expressing binding sites for I-B4. I-B4+ neurons require glial cell-derived neurotrophic factor (GDNF) and its receptor GFRα for trophic support. In GFRα null mutant mice, deficient innervation of the skin by I–B4+ neurons is accompanied by a decreased inflammatory pain response [47]. We found that S1P_1_ receptors are mainly expressed by I-B4+ nociceptors but are also present in some CGRP-positive neurons. Functionally, S1P_1_ receptor-dependent thermal hypersensitivity was partly mediated via TRPV1 receptors. Furthermore, activation of S1P_1_ receptors augmented the effects of TRPV1 receptor stimulation. This is an unexpected result since, in mice, only a small proportion of non-peptidergic I-B4+ neurons normally expresses the TRPV1 receptor [48]–[50]. However, exposure to protons massively increases the number of I-B4 neurons expressing TRPV1 receptors [51] and capsaicin induces ATF-3 translocation not only in peptidergic but also in I-B4+ non-peptidergic neurons [52]. Taken together, these observations indicate that functional TRPV1 and S1P_1_ receptors are probably co-expressed by subsets of peptidergic and non-peptidergic nociceptors in mice.

When applied intrathecally, S1P and FTY720 improve pain-related behavior in the formalin assay and modulate nociceptive processing via inhibition of neuronal cAMP synthesis at spinal cord level [32], [53]. This analgesic effect seems to oppose the pronociceptive effects of S1P we found at peripheral nociceptors and somewhat resembles the situation of endocannabinoids in nociception: depending on their site of action agonists at cannabinoid receptor 1 (CB1) exert analgesic effects via CB1 receptors expressed at peripheral nociceptors [44] or proalgesic effects at spinal cord level where they mediate C-fiber induced heterosynaptic pain sensitization via CB1 receptors expressed on spinal neurons [54]. Similar differential signaling pathways may apply for S1P and its receptors expressed on primary afferent nociceptors or spinal neurons.

To date it is unknown which of the S1P receptors mediate the spinal inhibitory effect. We generated mice lacking the S1P_1_ receptor in Na_v_1.8 expressing primary afferent nociceptors. These mice are largely resistant to S1P-induced hypersensitivity and to inflammatory thermal hypersensitivity. Together with data using S1P_1_ agonist SEW2871 we prove evidence that the proalgesic action of S1P on nociceptive nerve terminals is largely mediated by S1P_1_ receptors expressed by a subpopulation of sensory neurons. The remaining mild hypersensitivity may be explained by either S1P effects on non-neuronal S1P receptor expressing cells which in turn may release proalgesic substances. Second, SNS-Cre recombination only occurs in 90% of Na_v_1.8 positive neurons [45]. The few neurons still expressing S1P_1_ or nociceptors expressing other S1P receptors may also account for the remaining S1P effects.

This study provides evidence that S1P exerts a peripheral pronociceptive action by targeting neuronally expressed S1P_1_ receptors. The concept that S1P not only regulates inflammation but simultaneously tunes pain sensitivity is intriguing and suggests that S1P could contribute to conditions of chronic inflammatory pain like RA. If so, the development of antagonists simultaneously targeting S1P_1_ receptors in hyperreactive immune cells and hypersensitive peripheral nociceptors would be of great clinical benefit.

## Materials and Methods

### Ethic statement

All animal experiments have been performed with permission of the Austrian BMWF ministry (BMWF-66.011/0113-II/3b/2010; BMWF-66.011/0051-II/10b2008; GZ 66.011/85-C/GT/2007) and according to ethical guidelines of the IASP (International Association for the Study of Pain).

### Behavioral test

Male C57BL/6J mice (>8 weeks old) from an inbred colony were used in the experiments. TRPV1^-/-^ mice were a generous gift of J.B. Davis, GSK, Harlow, UK. Mice were housed on a 12 h light/dark cycle with free access to mouse chow and water. Standard testing procedures were used to quantify signs of pain-like behavior reflected by changes in thermal sensitivity. The area tested was the plantar side of the hind paw. Compounds were injected intracutaneously in a total volume of 5 µl. The experimenter was unaware of the nature of the treatment. Baseline measurements were taken twice on two days before and after injection for acute changes in heat sensitivity up to 24 hours post injection. Mice were placed in a plastic chamber and allowed to habituate for at least one hour. Heat sensitivity was assessed using the Hargreaves test [30]. A radiant heat source which delivered an increasing heat stimulus was focused on the plantar surface of the hind paw; the time from initiation of the radiant heat until paw withdrawal (paw withdrawal latency) was measured automatically (Ugo Basile, Italy). Each paw was tested three times and mean withdrawal latency was calculated. The interval between two trials on the same paw was at least one minute. All mice were maintained under SPF conditions. Littermates were used in all experiments to control for background effects and all animal use procedures were in accordance with ethical guidelines and animal welfare standards according to Austrian law. All behavioral measurements were done in awake, unrestrained, age-matched mice that were more than 8 weeks old by individuals who were blinded to the genotype of the mice being analyzed. The swelling of the hind (frontal and sagittal diameters) paw due to inflammation was measured after 48 h using a digital Vernier micrometer.

### Skin-nerve preparation and single fiber recordings

An *in vitro* skin-nerve preparation was used to investigate the properties of cutaneous afferent nerve fibers as previously published [55], [56]. Briefly, the preparation was superfused (15 ml/min) with modified synthetic interstitial solution containing (in mM) 108 NaCl, 3.48 KCl, 3.5 MgSO_4_, 26 NaHCO_3_, 1.7 NaH_2_PO_4_, 2.0 CaCl_2_, 9.6 sodium gluconate, 5.5 glucose, 7.6 sucrose saturated with oxygen with a temperature of 31±1°C and pH 7.4±0.05. Action potentials of single sensory neurons were recorded extracellularly from fine filaments dissected from the saphenous nerve, amplified (5000 fold), filtered (low pass 1 kHz, high pass 100 Hz), visualized on an oscilloscope and stored on a PC-type computer with the Spike/Spidi software package [57]. The fibers were characterized as unmyelinated (C) according to their conduction velocity and the receptive field was identified by mechanical probing of the skin with a glass rod. Standard heat stimuli with linear rise of the intracutaneous temperature (from 31±1°C to 47°C) were applied. A fiber was considered heat-sensitive if three or more action potentials were evoked during the stimulus. The heat threshold was either defined as the temperature that elicited the third spike of the response or as the temperature that evoked an instantaneous frequency of >1 imp/s. Both measures gave identical results. In order to prevent contamination of the entire preparation, chemical stimulation with S1P was restricted to a small area around the receptive field of a fiber by positioning a self-sealing metal cylinder around the receptive field.

### DRG neuron culture

Lumbar dorsal root ganglia (DRG) with the cell bodies of primary afferents that project into the hind paw were harvested from adult C57BL/6J mice, treated enzymatically with collagenase (Liberase®, Roche) and trypsin-EGTA (Invitrogen), and dissociated mechanically with a fire-polished Pasteur pipette as previously published [58], [59]. The resulting cell suspension was washed, plated on glass coverslips coated with poly-L-lysine/laminin (Sigma) and cultivated in synthetic serum-free medium (supplemented TNB™, Biochrom, Vienna) at 37°C in 5% CO_2_.

### Patch-clamp recordings

Using the whole-cell voltage-clamp configuration of the patch-clamp technique, ionic currents were recorded from isolated neurons at −80 mV holding potential after 18–32 hours as previously published [58], [59]. The external solution (ECS) contained (in mM): 145 NaCl, 5 KCl, 2 CaCl_2_, 1 MgCl_2_ (all Sigma), 10 glucose and 10 HEPES (Merck, Darmstadt, Germany), at pH 7.3 adjusted with NaOH (Merck). Borosilicate glass micropipettes (Science Products, Hofheim, Germany) pulled with a horizontal puller (Sutter Instruments Company, Novato, CA, USA) were filled with internal solution (ICS, in mM): 148 KCl, 2 MgCl_2_, 2 Na-ATP, 0.2 Na-GTP, 0.1 CaCl_2_, 1 EGTA (all Sigma) and 10 HEPES (Merck), at pH 7.3 adjusted with KOH (Merck). After filling, electrode resistance was 4–6 MΩ. Currents were filtered at 2.9 kHz, sampled at 3 kHz and recorded using an EPC-9 (HEKA, Germany) and the Pulse v8.74 software (HEKA). Experiments were performed at room temperature and only one neuron was tested per Petri dish. An automated seven-barrel system with common outlet at 100 µM distance to the cell under investigation was used for fast drug administration and heat stimulation [60]. Heat-activated inward currents (I_heat_) were elicited by applying ramp-shaped heat stimuli at 60 s intervals (linear temperature increase from 25 to 50°C within 5 s). S1P (0.1 to 10 µM, Sigma) or SEW2871 (0.01 to 10 µM, Sigma) were used as intermittent conditioning stimuli (60 s) followed by a 3-minute washout. Capsaicin was purchased from Sigma Aldrich.

### Tissue preparation

Specimens for RT-PCR and immunohistochemistry were obtained from 6 to 10-week-old C57BL/6J mice of either sex. DRG were snap-frozen in melting isopentane and stored at −80°C until required for immune staining/ISH or snap-frozen in liquid nitrogen for RT-PCR. For analysis of cultured neurons, 50 µl aliquots containing acutely dissociated cells (n = 6 mice) were used for subsequent qRT-PCR analysis. After plating on glass-bottom Petri dishes coated with poly-L-lysine (200 ng/ml, Sigma) cultures were kept at 37°C in a humid atmosphere containing 5% CO_2_ for 24 h. Cells grown on individual coverslips (n = 6 mice) were lysed and used for subsequent qRT-PCR analysis.

### Quantitative real-time PCR

Quantitative real-time PCR (RT-qPCR) was used to quantify levels of mRNAs using cDNA from thoracolumbar DRG, acutely dissociated and cultured DRG neurons (Corbett Roto-cycler, Sydney, Australia). DRG were lysed in RLT® buffer using a tissue lyser (Qiagen Doncaster, Australia), RNA was extracted (RNeasy Mini-kit, Qiagen) and the quantity and quality of the RNA determined (Nanodrop, Thermo Scientific, USA, Bioanalyzer, Agilent, USA). Acutely dissociated and primary cultures of DRG were lysed on the coverslip (RLT® buffer, Qiagen) and subsequently used for RNA extraction according to the manufacturer's instructions (RNeasy Micro-kit, Qiagen). The extraction was followed by DNase digestion and reverse transcription (Sensiscript, Qiagen). The cDNA was used for subsequent qPCR. All PCR reactions were prepared in triplicate from DRG of four to six animals using a ready-to-use kit according to the manufacturer's protocol (iQ SYBR green Supermix, Bio-Rad). Primers specific for mRNA sequences were designed using http://www.ncbi.nlm.nih.gov/tools/primer-blast/index.cgi?LINK_LOC=BlastHomeAd.

Primers specific for the reference gene, mouse HPRT, were used for standardization (Table 2). The efficiency of each primer pair was determined (between 0.96 and 1.1). Data were normalized by subtracting the threshold cycle (CT) levels between the genes of interest and HPRT [61]. The ΔCT values were subtracted from 50 showing higher values with higher expression. Amplicons were identified by sequencing (SA Pathology and Flinders Sequencing Facility).

**Table 2:** Primer pairs used.

Name	GI no.	Forward primer (5′-3′)	length (bp)
		Reverse primer (5′-3′)	
hypoxanthine guanine phosphoribosyl transferase (HPRT)	53237103	GCCCCAAAATGGTTAAGGTT	208
		TTGCGCTCATCTTAGGCTTT	
S1P1 = Edg1	NM_007901.3	CTCTGCTCCTGCTTTCCATC	173
		GCAGGCAATGAAGACACTCA	
S1P2 = Edg5	NM_010333.2	TCTCAGGGCATGTCACTCTG	162
		CAGCTTTTGTCACTGCCGTA	
S1P3 = Edg3	NM_010101.2	GTGTGTTCATTGCCTGTTGG	208
		TTGACTAGACAGCCGCACAC	
S1P4 = Edg6	NM_010102.1	GGCTACTGGCAGCTATCCTG	213
		GGAGGACGTGGAGACTTCTG	
S1P5 = Edg8	NM_053190.1	GATCCCTTCCTGGGTCTAGC	222
		TAGAGCTGCGATCCAAGGTT	

### Immunohistochemistry

DRG were serially cryosectioned at a thickness of 12 µm, fixed in Zamboni's fixative and subsequently preincubated for 1 h with PBS containing 10% normal donkey serum, 0.1% BSA, and 0.5% Tween 20. Indirect immunofluorescence was performed by overnight incubation with mixtures of the primary antisera (Table 3) at room temperature followed by washing in PBS and subsequent incubation with appropriate combinations of secondary reagents (Table 3) for 1 h at room temperature. The antiserum specifically recognizing S1P_1_ receptors [46] was a generous gift of S. Mandala, Immunology, Merck Research Laboratories, Rahway, New Jersey, USA. Preabsorption controls using the corresponding peptide (0.1 µg/l peptide) abolished immunolabeling (Fig. 3F). After incubation with the secondary reagents, the slides were washed in PBS and coverslipped in carbonate-buffered glycerol at pH 8.6. The slides were evaluated by sequential scanning using a confocal laser scanning microscope (TCS SP5, Leica, Bensheim, Germany). For quantification studies, two random ×40 magnification, non-overlapping confocal images from two sections from L2-L5 DRG (n = 5 animals) were taken. The longest and shortest diameter of neuronal profiles with a clearly visible nucleus were determined and the average diameter calculated. To assess double labeling, the neurons with clearly visible nuclei displaying a clear fluorescent signal were counted as immunoreactive.

**Table 3:** Primary and secondary antisera.

Antigen	Host	Dilution	Source
**Pimary antisera**			
S1P_1_	rabbit	1∶100	S. Mandala
Nf200	mouse, clone N52	1∶1000	Sigma
Peripherin	mouse, MAB 1527	1∶500	Chemicon
CGRP	goat	1∶1000	code1780, Arnel, New York, USA
I-B4	Bandeira simplicifolia	1∶1000	Sigma
**Secondary antisera**			
Cy3 anti rabbit Ig	donkey	1∶100	Jackson
Cy5 anti mouse Ig	donkey	1∶50	Jackson
AMCA anti mouse Ig	donkey	1∶50	Jackson
Cy5 anti sheep Ig	donkey	1∶25	Jackson

### Genetically modified mice

Mice homozygous for the floxed exon 2 allele of the mouse S1P_1_ receptor gene (*S1P_1_* ^fl/fl^), which encodes for the entire receptor protein, have been described previously [42] and were a generous gift of R. Proia. *S1P_1_* ^fl/fl^ mice were cross bred with *SNS-Cre* mice [43] to obtain homozygous *SNS-Cre:S1P_1_* ^fl/fl^ (hereinafter referred to as SNS-S1P1^−/−^) and *S1P_1_* ^fl/fl^ mice (control littermates; the recombinase is homo- or heterozygous). For detecting the wild type, knock-out and conditional alleles the following primers were used: 5′-GAGCGGAGGAAGTTAAAAGTG-3′, 5′-CCTCCTAAGAGATTGCAGCAA-3′ and 5′-GATCCTAAGGCAATGTCCTAGAATGGGACA-3′, all for S1P_1_, and primers 5′-GAAAGCAGCCATGTCCAATTTACTGACCGTAC-3′ and 5′-GCGCGCCTGAAGATATAGAAGA-3′ to detect SNS-Cre transgene expression. *SNS-Cre* mice, SNS-S1P_1_^-/-^ mice and their corresponding littermates (wild-type and *S1P_1_* ^fl/fl^ mice) had the genetic background C57BL/6J. For genotyping with quantitative real-time PCR, total RNA was isolated from DRG neurons immediately after preparation using TRI Reagent™ (Sigma) according to the manufacturer's instructions. Quality and degradation of RNA were controlled by denaturating agarose gel electrophoresis. RNA was treated with DNase I (Fermentas GmbH, St. Leon-Rot, Germany). Reverse transcription to cDNA was carried out using the GeneAmp RNA PCR Kit (Applied Biosystems). Each cDNA sample (100 ng) was analyzed for expression of S1P_1_ by quantitative real-time PCR using the TaqMan 5′ nuclease assays Mm00514644_m1 and Mm01352363_m1 (Succinate Dehydrogenase Subunit A, SDHA). Reactions were performed in a MicroAmp™ Fast Optical 96-Well Reaction Plate (Applied Biosystems) using the 7500 Fast Real-Time PCR System (Applied Biosystems) for thermal cycling and real-time fluorescence measurements. The PCR cycle protocol consists of 10 min at 95°C, and 40 two-step cycles of 15 sec each at 95°C and of 1 min at 60°C. Positive and negative controls were included in all experiments and each sample was run in triplicate for each PCR. Threshold cycle (C_T_) values were recorded as a measure of initial template concentration. Relative fold changes in RNA levels were calculated by the ΔΔC_T_ method using SDHA as a reference standard [62]. The range for the target, relative to a calibrator sample was calculated by 2^-ΔΔCT^.

### Microfluorimetric calcium measurements

Cells were plated on glass-bottom dishes coated with poly-L-lysine hydrobromide [10 µg/ml] and laminin [10 µg/ml] (both Sigma). Cultured cells were loaded with fura-2/AM (3 µM in ECS) and incubated for 30 min at 37°C in 5% CO_2_, washed twice with PBS (PAA) and used for experiments after an extra incubation of 30 min. Experiments were performed using an Axiovert microscope (Zeiss, Stuttgart, Germany) with a 40x/1,3 oil objective (Zeiss). Fura-2 was excited with a Xe-lamp at 340 nm and 380 nm (exposure time: 50 ms) with a polychrome IV monochromator (TILL Photonics GmbH, Munich, Germany), fluorescence intensity changes were recorded with a CCD camera (TILL Photonics) at 510 nm to determine fluorescence intensity ratios at this wavelength as previously published [63]. For data acquisition MetaFluor4.6r8 (Universal Imaging Corp., Franklin, USA) was used and off-line analysis was performed with OriginPro7.SR2 (Origin Lab Corporation).

### Statistical analysis

For detailed statistical analysis the SigmaStat 3 software package was used. Data are presented as mean ± s.e.m. Statistical tests were used depending on sample size, distribution and number of variables: One- or two-way ANOVA followed by post hoc test, the non-parametric Mann-Whitney U-test or the parametric Student's unpaired t-test for comparison between groups; for intra-individual comparisons, the Wilcoxon matched pairs test or the parametric Student's paired t-test was used. Differences were considered statistically significant at p<0.05.
